# Theoretical Study of 2-(Trifluoromethyl)phenothiazine Derivatives with Two Hydroxyl Groups in the Side Chain-DFT and QTAIM Computations

**DOI:** 10.3390/molecules26175242

**Published:** 2021-08-29

**Authors:** Andrzej Poła, Anna Palko-Łabuz, Kamila Środa-Pomianek

**Affiliations:** Department of Biophysics and Neurobiology, Wroclaw Medical University, 50-368 Wrocław, Poland; andrzej.pola@umed.wroc.pl (A.P.); kamila.sroda-pomianek@umed.wroc.pl (K.Ś.-P.)

**Keywords:** DFT theory, QTAIM theory, phenothiazines, antioxidant

## Abstract

Phenothiazines are known as synthetic antipsychotic drugs that exhibit a wide range of biological effects. Their properties result from the structure and variability of substituents in the heterocyclic system. It is known that different quantum chemical properties have a significant impact on drug behavior in the biological systems. Thus, due to the diversity in the chemical structure of phenothiazines as well as other drugs containing heterocyclic systems, quantum chemical calculations provide valuable methods in predicting their activity. In our study, DFT computations were applied to show some thermochemical parameters (bond dissociation enthalpy—BDE, ionization potential—IP, proton dissociation enthalpy—PDE, proton affinity—PA, and electrontransfer enthalpy—ETE) describing the process of releasing the hydrogen/proton from the hydroxyl group in the side chain of four 2-(trifluoromethyl)phenothiazine (TFMP) derivatives and fluphenazine (FLU). Additional theoretical analysis was carried out based on QTAIM theory. The results allowed theoretical determination of the ability of compounds to scavenge free radicals. In addition, the intramolecular hydrogen bond (H-bond) between the H-atom of the hydroxyl group and the N-atom located in the side chain of the investigated compounds has been identified and characterized.

## 1. Introduction

Quantitative structure–activity relationship (QSAR) techniques provide a valuable approach to establishing a direct relationship between the quantum chemical properties and the biological activities of chemical compounds. QSAR studies include computational tools showing molecular parameters of the compounds that may predict their usage in drug discovery [1]. Except from QSAR analysis, the biological properties can be theoretically assessed by calculation of the thermochemical parameters describing the physicochemical characteristic of different chemical groups in the structure of compounds. The density functional theory (DFT), popular quantum chemical method, allows for determine theoretical molecular stability as well as chemical reactivity [2]. On the other hand, the quantum theory of atoms in molecules (QTAIM) describes atomic and bond properties based on molecular electron-density distributions [3]. Both DFT and QTAIM have been used in theoretical investigations on different compounds with anticancer activity [4,5,6]. Using these theories, the activity of the chemical compounds may be explained in terms of the predicted chemical reactivity descriptors as well as molecular electrostatic potential. DFT and the molecular docking studies of Al Sheikh Ali et al. [4] confirmed the results of in vitro experiments on anticancer activity of triazoles derivatives against Caco-2, HCT116, HeLa, and MCF7 cell, indicating the most active among studied compounds. Mary et al. [5] used DFT theory to investigate the molecular geometries, vibrational spectra, electronic properties, and molecular electrostatic potential of pharmaceutically active chromone derivatives. The results of these calculations, together with the QTAIM studies, allowed for the conclusion that the studied compounds possess higher anticancer activity as compared with antitumor standards (5-fuorouracil and azathioprine). Karelou et al. [6] used computational chemistry experiments in order to show the instability of some novel aza-acridine aminoderivatives in aqueous media, which leads to the moderate anticancer activity of these compounds.

**Figure 1:**
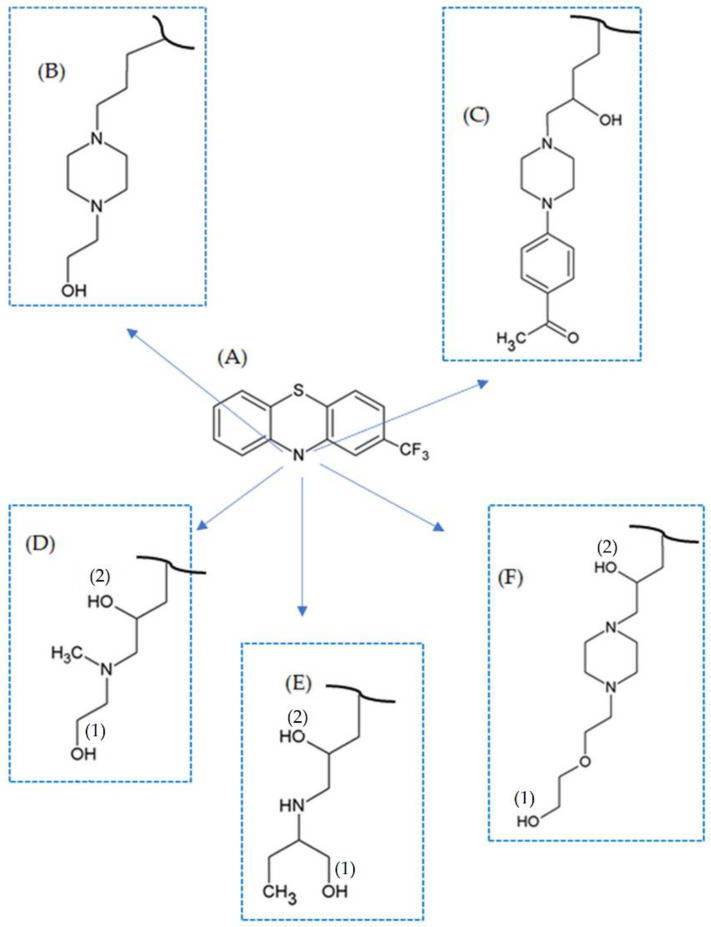
2-(Trifluoromethyl)phenothiazine, TFMP (**A**), and structure of the side chain for its derivatives: FLU (**B**), APh-FLU (**C**), MAE-TPR (**D**), ABu-TPR (**E**), and HEE-FLU (**F**).

In our research, we studied theoretically four newly synthesized 2-(trifluoromethyl)phenothiazine (TFMP) derivatives: (10-[3-(*N*-2-hydroxyethyl-*N*-methylamino)-2-hydroxypropyl]-2-trifluoromethylphenothiazine) (MAE-TPR), (S(+)-10-[3-(1-ethyl-2-hydroxyethylamino)-2-hydroxypropyl]-2-trifluoromethylphenothiazine hydrochloride) (ABu-TPR), (10-{3-[4-(2-hydroxyethoxyethyl)piperazin-1-yl]-2-hydroxypropyl}-2-trifluoromethylphenothiazine dihydrochloride) (HEE-FLU), and (10-{3-[4-(4-acetylphenyl)piperazin-1-yl]-2-hydroxypropyl}-2-trifluoromethylphenothiazine dihydrochloride) (APh-FLU), and fluphenazine (FLU) (Figure 1A–E). Our previous in vitro research revealed that these compounds possess pro-oxidative and pro-apoptotic activity against colon cancer cells, and they are also sensitive and resistant to the drug doxorubicin [7]. Here, the study was mainly concerned with the quantum chemical properties of the hydroxyl groups located in the side chain attached to the nitrogen atom at position 10 (N10) of the TFMP three-membered ring. In the side chain of FLU, this group was located at the end of the side chain, while in the case of APh-FLU, the hydroxyl group was present near the three-membered ring. On the other hand, in the side chain of MAE-TPR, ABu-TPR, and HEE-FLU, there were two hydroxyl groups, one of them at the end of the side chain, and the other near the TFMP three-membered ring. These hydroxyl groups can release the hydrogen atom or a proton by different chemical reactions, i.e., homolytic hydrogen atom transfer (HHAT), single-electron transfer–proton transfer (SET-PT), or sequential proton loss electron transfer (SPLET). The mechanisms mentioned above may co-exist. They are the consequence of solvent properties and radical characteristics. Each of these chemical schemes is described by appropriate thermochemical parameters: bond dissociation enthalpy (BDE), ionization potential (IP), proton dissociation enthalpy (PDE), proton affinity (PA), and electron transfer enthalpy (ETE). It has been established that the bond dissociation enthalpy (BDE) of the O–H group is associated with the HHAT mechanism of free radical scavenging that is promoted in gas-phase and non-polar solvents. However, the BDE can be also used to describe the radical scavenging processes occurring in polar solvents (SET-PT and SPLET). This is due to the fact that the total energy requirements associated with the SET-PT-sum of the IP and PDE—as well as the SPLET-sum of the PA and ETE—perfectly correspond with the BDE. The values of these parameters were calculated from the total enthalpy of a given derivative and its different chemical forms, i.e., anion, radical, and cationic radical, using DFT/B3LYP/6-311++G(d,p) and CPCM/DFT/B3LYP/6-311++G(d,p) methods [8], in the gaseous phase and the aqueous medium, respectively. Our results revealed that from the theoretical point of view, the studied compounds possess antioxidative properties. Moreover, the QTAIM analysis allowed for the identification of the intra-molecular hydrogen bond (H-bond) between the H-atom of the hydroxyl group and the N-atom located in the side chain.

## 2. Results and Discussion

### 2.1. DFT Calculations at the Ground State in the Gaseous Phase

The values of the BDE, IP, PDE, PA, and ETE parameters for the hydroxyl groups located in the side chain of the examined TFMP derivatives are collected in Table 1. FLU and APh-FLU are derivatives with one hydroxyl group in the side chain. In the case of FLU, this group is located at the end of this chain, whereas in APh-FLU, this group is located closer to the TFMP three-membered ring. For such a location of the APh-FLU derivative hydroxyl group, the BDE, PDE, and PA parameters were lower by 0.8 kcal × mol^−1^, 6.1 kcal × mol^−1^, and 20.9 kcal × mol^−1^ than values calculated for the hydroxyl group located at the end of the side chain of FLU. The relatively small difference between values of the BDE parameter showed that from the thermodynamic point of view, the location of the hydroxyl group in the side chain did not matter for the probability of participation of this group in the HHAT reaction. On the other hand, the value of the BDE parameter for derivatives both of FLU (99.1 kcal × mol^−1^) and APh-FLU (98.3 kcal × mol^−1^) was over two times and over three times lower than the value of PDE (257.7 kcal × mol^−1^ for FLU and 251.6 kcal × mol^−1^ for APh-FLU) and PA (369.6 kcal × mol^−1^ for FLU and 348.7 kcal × mol^−1^ for APh-FLU) parameters, respectively. The relatively very large values of these two parameters describing the SET-PT and SPLET chemical mechanisms indicate that in the gaseous phase, heterolytic cleavage of the OH bond is less likely than homolytic rupture of this bond (HHAT mechanism). This is probably related to the fact that in the gaseous phase, the formation of electrically uncharged products due to the homolytic OH bond cleavage is thermodynamically favorable [9,10,11]. Analogical relations were observed between the values of BDE, PDE, and PA parameters for the newly synthesized derivatives of MAE-TPR, ABu-TPR, and HEE-FLU. The values of the mentioned parameters for these three derivatives were lower for that hydroxyl group, which was located closer to the TFMP three-membered ring. Moreover, for both OH groups, the values of PDE and PA were several times higher than for the BDE parameter. These data lead to two conclusions. First, in the gaseous phase, the chemically active place is the OH group located closer to the TFMP three-membered ring, and second, this OH group is theoretically more likely to undergo the HHAT reaction. Considering only these three newly synthesized derivatives, their antioxidative properties can be arranged in the following order: MAE-TPR > ABu-TPR > HEE-FLU.

### 2.2. DFT Calculations at the Ground State in the Aqueous Medium

From a biological point of view, antioxidants fulfill their role in the physiological liquids of living systems. Therefore, the values of thermochemical parameters for an aqueous medium are very important (Table 1). In this medium, the BDE parameter decreased its value by several kcal × mol^−1^ compared to its value for the gaseous phase. On the other hand, the value of other parameters, i.e., IP, PDE, and PA, decreased drastically in the aqueous medium compared to their value in the gaseous phase, about twice for IP parameters and several times for the PDE and PA parameters. Compared to the gaseous phase, for the aqueous medium, a slight decrease in the calculated value of the BDE parameter and a several-fold decrease in the calculated values of the IP, PDE, and PA parameters were observed for the hydroxyl groups found in isoflavones [10], hydroxycoumarin derivatives [12], chalcones [13], and flavonoids [14]. For derivatives with one hydroxyl group in the side chain, the value of the BDE parameter was 94.3 kcal × mol^−1^ in the case of the FLU side chain (group –OH(1)), while this value was lower and equal to 92.8 kcal × mol^−1^ in the case of the APh-FLU side chain (group –OH(2) located closer to the three-membered ring). Similarly, the PDE and PA parameters were higher for the hydroxyl group located in the side chain of FLU (PDE = 53.3 kcal × mol^−1^; PA = 64.1 kcal × mol^−1^) than for the side chain of APh-FLU (PDE = 46.4 kcal × mol^−1^; PA = 60.1 kcal × mol^−1^) (Table 1). These data indicate that the O-H bond in the hydroxyl group located closer to the TFMP three-membered ring can potentially break more easily. For the newly synthesized TFMP derivatives possessing two hydroxyl groups in the side chain (MAE-TPR, ABu-TPR, and HEE-FLU), analogous relations between the value of thermochemical parameters were also observed. The calculated value of the BDE parameter was higher for the –OH(1) group than for the −OH(2) group. It can be concluded that the –OH(2) group is the active site in the molecules of these derivatives. The value of BDE for–OH(2) was the highest (93.1 kcal × mol^−1^) for the HEE-FLU derivative and the lowest (84.6 kcal × mol^−1^) for the MAE-TPR derivative. Considering the calculated value of the BDE parameter for the –OH(2) group located in the side chain of the MAE-TPR, ABu-TPR, and HEE-FLU, it can be concluded that the theoretical obtained antioxidative activity of these derivatives for the HHAT mechanism can be ranked in the following order: MAE-TPR > ABu-TPR > HEE-FLU.

In the SET-PT mechanism, the first chemical reaction is associated with the formation of a cationic radical due to the transfer of an electron from the molecule to the environment. This process is thermodynamically described by the IP (ionization potential) parameter. For the newly synthesized derivatives, this parameter in the aqueous medium was the highest (87.6 kcal × mol^−1^) for MAE-TPR and was the lowest (86.7 kcal × mol^−1^) for HEE-FLU. The slight difference between these values of IP (0.9 kcal × mol^−1^) might indicate that the ionization process of a molecule is not very important for the second chemical reaction in the SET-PT mechanism. This second reaction is related to the releasing of a proton from the hydroxyl group of the cationic radical and is described by the PDE thermochemical parameter. Similar to the BDE parameter, the value of the PDE parameter for MAE-TPR, ABu-TPR, and HEE-FLU was higher for the –OH(1) group than for the –OH(2) group, confirming that in the SET-PT mechanism, the –OH(2) group is an active site for potential reactions with free radicals. For the –OH(2) group, the value of the PDE parameter in an aqueous medium was the highest (46.7 kcal × mol^−1^) for HEE-FLU and the lowest (37.2 kcal × mol^−1^) for MAE-TPR. These data indicate that in the SET-PT mechanism, the theoretical antioxidative properties of the newly synthesized TFMP derivatives can be arranged in the following order MAE-TPR > ABu-TPR > HEE-FLU.

The first step in the SPLET chemical path is a heterolytic cleavage of the hydroxyl group. This reaction is described by the PA thermochemical parameter, which determines the ability of the molecule to accept a proton. On the other hand, the ability of a given hydroxyl group to form the anionic form (loss a proton) of MAE-TPR, ABu-TPR, and HEE-FLU derivatives can be also determined by this parameter. The value of the PA parameter for MAE-TPR and ABu-TPR was lower for the –OH(2) group as compared to the value of this parameter for the –OH(1) group. In case of the HEE-FLU derivative, the value of the PA parameter for both hydroxyl groups was practically the same (60.9 kcal × mol^−1^ for –OH(2) and 60.8 kcal × mol^−1^ for –OH(1) group, respectively). These data indicated that in the contrast to MAE-TPR and ABu-TPR, in an aqueous medium, both hydroxyl groups in the side chain of the HEE-FLU derivative undergo the SPLET mechanism with the same probability. In the SPLET mechanism, a radical is formed in the second reaction due to electron transfer from the anionic form of the molecule to the environment. This process is described by the thermochemical parameter ETE. For both hydroxyl groups, the values of this parameter differed only slightly (Table 1). This means that the formation of radicals in the SPLET chemical path depends mainly on the occurrence of the first reaction. For the –OH(2) group, the value of the PA parameter describing energetically the first reaction in the SPLET mechanism was the lowest for MAE-TPR and the highest for HEE-FLU. Analogously to the HHAT and SET-PT mechanisms, the antioxidative properties of these newly synthesized compounds in the SPLET mechanism can be arranged as follows: MAE-TPR > ABu-TPR > HEE-FLU. These three newly synthesized derivatives of TFMP were investigated [7] as the free radicals’ scavengers in the sensitive for chemotherapy LoVo cell line as well as the drug-resistant LoVo/Dx cell line. In this study, the intracellular level of the reactive oxygen species (ROS) was estimated by the fluorescence method. In the cells of both lines, the ROS level was the lowest at the presence of the MAE-TPR derivative and the highest at the presence of the HEE-FLU derivative. This result implies that the free radical scavenging efficiency of the tested compounds is MAE-TPR > ABu-TPR > HEE-FLU, which is consistent with the results of our theoretical calculations.

### 2.3. Quantum Theory of Atoms in Molecules (QTAIM)

**Figure 2:**
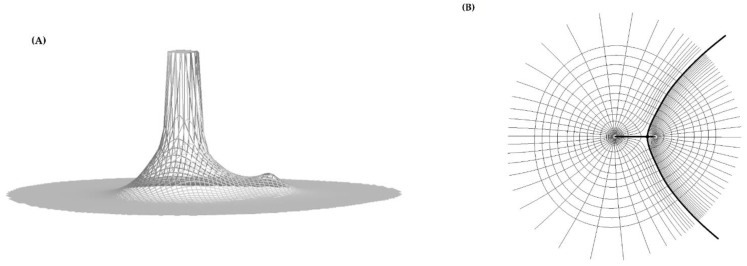
The relief map of electron density distribution in the hydroxyl group (**A**). The two-dimensional scheme of the division of the physical space on monoatomic subspaces occupied by the atoms of the hydroxyl group (**B**).

The QTAIM theory was proposed by R. F. W. Bader [15]. The QTAIM is a model of molecular and condensed matter electronic systems in which atoms and bonds are natural expressions of a system’s observable electron density distribution function (ρ) [16]. QTAIM allows the calculation of certain physical properties on a per-atom basis by dividing space up into atomic volumes containing exactly one nucleus, which acts as a local attractor of the electron density. The distribution of the electron density function for the hydroxyl group (as an example) is shown on the left in Figure 2.

This distribution exhibits two maxima and the saddle part between these maxima. The topological analysis of ρ works by calculation of the gradient (∇ρ) of this function. If ∇ρ = 0, it is possible to designate the location of the critical points for this function. Two quantities characterize the critical points of ρ: rank (r) and signature (s), which are written as (r, s) [17]. For the stable topological critical points, the rank r = 3. Signature refers to the sum of the signs of the eigenvalues of the electron density Hessian matrix. For an example of the hydroxyl group, each of two maxima is characterized by the rank and signature (3, −3) and is called the nuclear critical point (NCP). NCP corresponds to the attractor position of the gradient field. The saddle part on the electron density distribution picture corresponds to a potential chemical bond. This region of electron density is described by the bond critical point (BCP), for which the rank and signature are equal (3, −1), respectively. The electron density function’s topological analysis also enables the division of the physical space occupied by the molecule on monoatomic subspaces. To designate the disjointed three-dimensional areas for the given atom of the molecule, zero-flux surfaces are used. The given surface has met a condition expressed by equation in which the gradient vector is computed at the surface point where the normal vector is located [16].

(1) $$\nabla \rho \;\times \;\vec{n}=0$$

**Figure 3:**
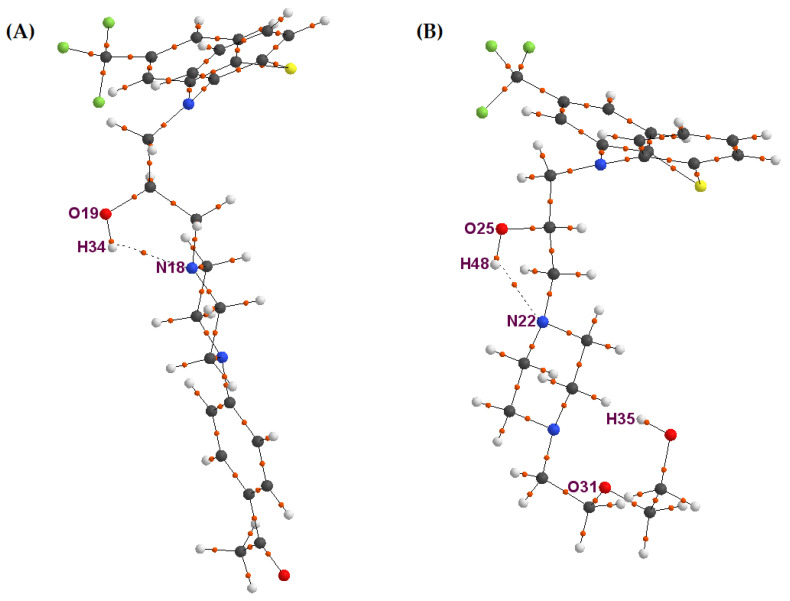
The molecular graphs for APh-FLU (**A**) and for HEE-FLU (**B**). The black straight lines and black dashed lines represent the bond paths between attractors. Attractors correspond to each atom’s position: carbon (black), hydrogen (white), nitrogen (blue), oxygen (red), and fluorine atoms (green). The orange smallest points represent the BCPs for the bond paths.

In this scalar product, ∇ρ stands for a gradient vector of electron density, and $\vec{n}$ stands for a normal vector to this surface. At the right in Figure 2, the two-dimensional topology for the hydroxyl group resulting from the QTAIM is shown. In this scheme, closed lines represent a map of the electron density of the hydroxyl group. The lines originate at infinity and terminate on the maxima of ρ, representing the gradient vector field of this function. The thick straight line represents a bond path (BP) between attractors, and the black point located on the bond path is a critical point (BCP) of this bond path. The thick hyperbola-shape line through BCP represents a zero-flux surface, which met a condition (1) and thus allows divides the physical space occupied by the hydroxyl group into two subspaces for each of the atoms of this group, for the oxygen at the left side and hydrogen at the right side. For all examined TFMP derivatives, the analysis of electron density function was performed using the AIMAll [18] application. Special attention was directed to the numerical parameters for the BCPs located on the BPs between the oxygen and hydrogen atoms in hydroxyl groups as well as BCPs located on the BPs between the hydrogen atom of the OH group and nitrogen atom located in the side chain. The molecular graphs for APh-FLU and HEE-FLU in the gaseous phase are shown in Figure 3. In these graphs, two kinds of bond paths can be observed. One of them is depicted as a black straight line, and the second is depicted as a black dashed line. The potential chemical bond between given atoms can be characterized on the basis of QTAIM. For this purpose, it is necessary to calculate the values of the numerical parameters for BCP, i.e.,: electron density (ρ_BCP_), Laplacian of electron density $\left(\nabla_{\varrho_{BCP}}^{2}\right)$, potential energy density ($V_{BCP}$), kinetic energy density ($G_{BCP}$), and total energy density ($H_{BCP}$). For a covalent bond, these parameters should meet the following relations: the value of $\varrho_{BCP}$ should be relatively large (from tenths to several atomic units), $\left|V_{BCP}\right|$<<0, $\nabla_{\varrho_{BCP}}^{2}$< 0, $G_{BCP\;}$<<$\left|V_{BCP}\right|$, and $H_{BCP}$ < 0 [19,20,21]. The values of these parameters for BCPs located on the bond paths between the oxygen and hydrogen atoms (straight black line) of the hydroxyl group are collected in Table 2.

**Figure 4:**
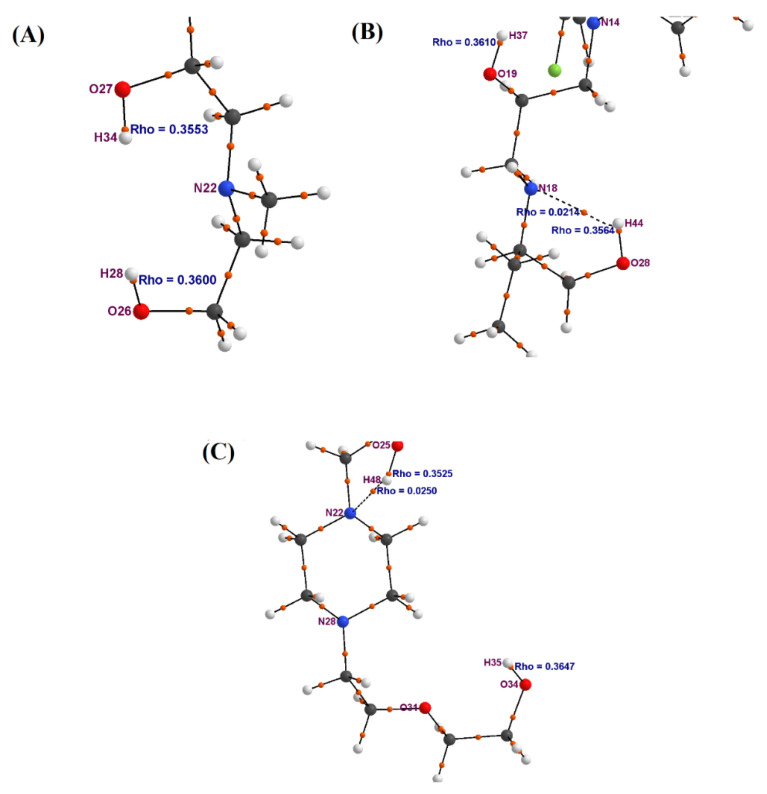
The molecular graphs of the side chain for MAE-TPR (**A**), Abu-TPR (**B**), and HEE-FLU (**C**) in aqueous medium. The electron density (Rho) values of the BCPs were calculated on the basis of the wave function, using AIMAll software.

These values meet the listed above criteria for all investigated TFMP derivatives and unambiguously show that these bond paths represent the covalent chemical bonds. The bond paths depicted by dashed lines can be observed in the gaseous phase for APh-FLU and HEE-FLU only. The value of electron density for the BCPs of the bond paths represented by dashed lines, i.e., between H34 and N18 atoms for APh-FLU and between H48 and N22 atoms for HEE-FLU, was ρ_BCP_ = 0.02037 and ρ_BCP_ = 0.02164, respectively (Table 3). These electron density values were several times lower than for the BCPs located on the bond paths, which represent the covalent bond. These low electron density values for the BCPs of these bond paths suggested that the dashed lines represent a different type of chemical bond from the covalent bond. It can be assumed that the dashed lines represent an H-bond. The value of numerical parameters for BCP lying on the bond path representing a potential H-bond should meet the following relations: $\varrho_{BCP\;}$ should be in the range of 0.002–0.034 (a.u.), $\nabla_{\varrho_{BCP}}^{2}$ > 0 and should be in the range of 0.024–0.139 (a.u.), $V_{BCP\;}$ < 0, $G_{BCP\;}\approx \left|V_{BCP}\right|$, and $H_{BCP}$ ≈ 0 [19,22]. These criteria are met by the calculated value (Table 3) of the numerical parameters for BCP located on the bond path depicted by the dashed line. These data for the gaseous phase showed that in the case of APh-FLU and HEE-FLU, the dashed lines represent the H-bond. The discussed H-bond was present for APh-FLU and HEE-FLU also in the aqueous medium and appeared additionally between N18 and H44 atoms for the ABu-TPR derivative. The molecular graphs of the side chain with two hydroxyl groups i.e., for MAE-TPR, ABu-TPR, and HEE-FLU for the aqueous medium, are present in Figure 4. 

These graphs showed that in contrast to the ABu-TPR and HEE-FLU derivatives, in the side chain of MAE-TPR, there are no bond paths that represent a potential H-bond. On the other hand, the DFT calculated data for the aqueous medium showed that from a theoretical point of view, the active place in the molecules of TFMP derivatives with two hydroxyl groups located in the side chain was the –OH(2) group. For these three derivatives, the difference between the values of the thermochemical parameters describing the physicochemical properties of the hydroxyl groups in their side chains was also calculated. For this purpose, the smaller value of a given parameter for one hydroxyl group was subtracted from the larger value of that parameter for the other hydroxyl group. The values of these differences (i.e., ΔBDE, ΔPDE, and ΔPA) are collected in Table 1.

This difference in the values of thermochemical parameters was the highest for the ABu-TPR derivative and was the lowest for the HEE-FLU derivative. A question may be asked at this point of whether the H-bond influenced the physicochemical properties of the specific hydroxyl group in the side chain of the TFMP derivatives. It seems that the QTAIM analysis can help in providing an answer to that question. In the case of the MAE-TPR derivative that lacked any H-bonds, the only theoretical factor to predestine the –OH(2) group as the chemically active place was the electron density between the oxygen and hydrogen atoms of this group. The lower the value of the electron density, the weaker the chemical bond and the easier this bond rupture. Indeed, the value of electron density between the O27 and H34 atoms of the –OH(2) group was lower than this value for the –OH(1) group, between O26 and H28 atoms (Figure 4 and Table 2). The electron-withdrawing –CF_3_ group located in the TFMP three-membered ring probably caused such distribution of the electron cloud in the side chain of this derivative. For this derivative, the value of the discussed difference between specific thermochemical parameters, i.e., ΔBDE, ΔPDE, and ΔPA was within the range of 1–2 kcal × mol^−1^. While for the ABu-TPR derivative, this difference was over two times larger and was within the range of 3–5 kcal × mol^−1^. In the case of ABu-TPR, the H-bond was theoretically created between the H34 atom of the –OH(1) group and the N18 atom of the side chain. This additional H-bond stabilized the bond of the H34 atom in the –OH(1) group, and as a consequence, it caused an increase in the difference between the specific thermochemical parameters that describe the physicochemical properties of both hydroxyl groups. In contrast to the case of ABu-TPR, for the HEE-FLU derivative, the additional H-bond was theoretically created between the H48 atom in the –OH(2) group and the N22 atom in the side chain. The stabilization of the bond of the H48 atom in the –OH(2) group by an additional H-bond caused a significant decrease in the difference between specific thermochemical parameters for both hydroxyl groups, and these values were within the range of 0.1–0.7 kcal × mol^−1^. The presented theoretical analysis based on the QTAIM calculations for MAE-TPR, ABu-TPR, and HEE-FLU of TFMP derivatives in the aqueous medium showed that creating an H-bond between the H atom of the hydroxyl group and the nitrogen atom located in the side chain facilitated or hindered the release of the proton or the hydrogen atom from a specific hydroxyl group.

Table 3 presents the values of the distance between atoms that may potentially form hydrogen bonds. These data indicate that the H-bond can be created if the distance between the H-atom of the hydroxyl group and the N-atom or O-atom in the side chain was equal or less than 2.22 Å and 2.19 Å respectively for the gaseous phase and the aqueous medium. To determine the H-bond strength ($E_{HB}$), the potential energy density ($V_{BCP}$) value of BCP for the hydrogen bond path can be used [23,24] (2).

(2) $$E_{HB}=\frac{1}{2}V_{BCP}$$

The data of the estimated value of H-bond energy is shown in Table 3. The values of the hydrogen bond energy were within 4.6–5.7 kcal × mol^−1^. According to the classification proposed by Rosaz [25], the H-bonds occurring in the side chain of the examined TFMP derivatives are classified as weak bonds. It was also noted that there exists a correlation between the energy of the H-bond and the value of electron density at the BCP and bond length. The large value of the electron density in the BCP of the bond path is connected with the large value of the bond energy, whereas the large value of the bond length is connected with the low value of the bond energy [18,26]. The numerical data calculated for the investigated TFMP and collected in Table 3 fully confirm these correlations for the aqueous medium. The biggest length of the H-bond and the lowest value of the electron density was for the ABu-TPR derivative, and the value of the H-bond energy for this derivative was simultaneously be the lowest.

## 3. Materials and Methods

### 3.1. Compounds

For the investigation, the following 2-(trifluoromethyl)phenothiazine (TFMP) derivatives were chosen: fluphenazine (FLU) and newly synthesized derivatives; MAE-TPR(10-[3-(*N*-2-hydroxyethyl-*N*-methylamino)-2-hydroxypropyl]-2-trifluoromethylphenothiazine), ABu-TPR (S(+)-10-[3-(1-ethyl-2-hydroxyethylamino)-2-hydroxypropyl]-2-trifluoromethylphenothiazine hydrochloride), HEE-FLU (10-{3-[4-(2-hydroxyethoxyethyl)piperazin-1-yl]-2-hydroxypropyl}-2-trifluoromethylphenothiazine dihydrochloride) and APh-FLU (10-{3-[4-(4-acetylphenyl)piperazin-1-yl]-2-hydroxypropyl}-2-trifluoromethylphenothiazine dihydrochloride). In the structure of the TFMP molecule, two aromatic rings are linked by sulfur and nitrogen atoms. This connection caused the creation of the three-membered structure with the middle hexagonal ring, including these two atoms. In position 2 of this ring, there is an electron-withdrawing –CF_3_ group (Figure 1). In the side chain of FLU and APh-FLU, there is one hydroxyl group, whereas in the case of newly synthesized derivatives MAE-TPR, ABu-TPR, and HEE-FLU, there are two hydroxyl groups. Regardless of the number of these groups in the side chain, the hydroxyl group located on the end of this chain was marked as–OH(1), whereas the hydroxyl group located closer to the TFMP three-membered ring was marked as –OH(2).

### 3.2. Computational Details

#### 3.2.1. Chemical Reactions

In the biological systems, the TFMP derivatives, whose molecules include the hydroxyl groups (TFMP–OH), can undergo chemical reactions that involve rupture of the chemical bond inside the hydroxyl group. These reactions can be divided into three groups [27,28]: Homolytic Hydrogen Atom Transfer (HHAT) ($TFMP-OH\to TFMP-O^{\cdot }+H^{\cdot }$), Single Electron Transfer-Proton Transfer (SET-PT) ($TFMP-OH\to TFMP-OH^{\cdot +}+e^{-}$; $TFMP-OH^{\cdot +}\to TFMP-O^{\cdot }+H^{+}$), and Single Proton-Loss Electron Transfer (SPLET) ($TFMP-OH\to TFMP-O^{-}+H^{+}$; $TFMP-O^{-}\to TFMP-O^{\cdot }+e^{-}$).

#### 3.2.2. Thermochemical Parameters

Each above reaction can be characterized by an appropriate parameter: BDE (bond dissociation enthalpy) for the HHAT mechanism, IP (ionization potential) and PDE (proton dissociation enthalpy) for the SET-PT mechanism, and PA (proton affinity), and ETE (electron transfer enthalpy) for the SPLET mechanism [27,29]. These thermochemical parameters can be calculated using the following equations [30,31].

(3) $$\mathrm{BDE}=H_{t}\left(TFMP-O^{\cdot }\right)+H_{t}\left(H^{\cdot }\right)-H_{t}\left(TFMP-OH\right)$$

(4) $$\mathrm{IP}=H_{t}\left(TFMP-OH^{\cdot +}\right)+H_{t}\left(e^{-}\right)-H_{t}\left(TFMP-OH\right)$$

(5) $$\mathrm{PDE}=H_{t}\left(TFMP-O^{\cdot }\right)+H_{t}\left(H^{+}\right)-H_{t}\left(TFMP-OH^{\cdot +}\right)$$

(6) $$\mathrm{PA}=H_{t}\left(TFMP-O^{-}\right)+H_{t}\left(H^{+}\right)-H_{t}\left(TFMP-OH\right)$$

(7) $$\mathrm{ETE}=H_{t}\left(TFMP-O^{\cdot }\right)+H_{t}\left(e^{-}\right)-H_{t}\left(TFMP-O^{-}\right)$$

*H_t_* is the total enthalpy, whose value was calculated according to Equation (8), for the molecule as well as for its different chemical forms, i.e., anion, radical, and cationic radical.

(8) $$H_{t}=E_{0}+ZPE+H_{trans}+H_{rot}+H_{vib}+RT,$$

where *E*_0_ expresses the total electronic energy, *ZPE* stands for zero-point energy, and *H_trans_*, *H_rot_,* and *H_vib_* are the translational, rotational, and vibrational contributions to the total enthalpy, respectively. To convert the total energy into a total enthalpy, the RT term that represents the *pV*-work was added. The quantities of the parameters described in the reaction mentioned above were calculated at 298.15 K and 1 atm.

#### 3.2.3. Computational Calculations

The value of total enthalpy in the gaseous phase for each molecule and specified chemical form, i.e., cationic radical, anion, and radical, was calculated using the SPARTAN ’18 package [32], according to the following procedure. In the first step, for a given molecule, the MMFF94 molecular mechanics model was used, and the series of aligned conformers were obtained, starting from the lowest total energy. Using the Boltzmann Weights (BW) option, we selected those conformers for which BW < 0.05. Next, for these conformers, the full optimization was performed using the HF/3-21G (Hartree–Fock) model. Finally, to refine the geometry optimization and analyze the vibrational frequencies, the lowest-energy conformer was selected, and calculations were performed using the DFT/B3LYP/6-311++G(d,p) (Density Functional Theory) method. The value of total enthalpy for other chemical forms was calculated from the fully optimized TFMP -OH molecule, using the same calculation method. The full optimization geometry in the aqueous medium of all chemical forms of the investigated TFMP was determined using the CPCM/DFT/B3LYP/6-311++G(d,p) (Conductor-like Polarizable Continuum Model) calculation method. The absolute minimum of the potential energy surface for each of the optimized structures was confirmed by vibration analysis; no imaginary frequencies were observed. For the gaseous phase calculations, we used earlier determined total enthalpy values for the electron $H_{t}\left(e^{-}\right)$ = 0.00119 h (hartree) [33], for the proton $H_{t}\left(H^{+}\right)$ = 0.00236 h [34,35], and for the hydrogen atom $H_{t}\left(H^{\cdot }\right)$= −0.49764 h [36]. However, for chemical reactions for the HAT, SET-PT, and SPLET mechanisms in the aqueous medium, the values of the hydration enthalpy of the species $H^{+}$, $H^{\cdot }$, and $e^{-}$ are not available, and their theoretical quantity depends on the quantum model used. For the determination of these values, the model proposed by Rimarcik [30] was used. The hydration enthalpy for these species was calculated using the CPCM/ DFT/B3LYP/6-311++G(d,p) model, and the values of the total enthalpy of $H^{+}$, $H^{\cdot }$, and $e^{-}$ for the aqueous environment used in this paper are equal: $H_{t}\left(e^{-}\right)\;$= −0.045102 h, $H_{t}\left(H^{+}\right)\;$= −0.38634 h, $H_{t}\left(H^{\cdot }\right)\;$= −0.499164 h. In the side chain of MAE-TPR, ABu-TPR, and HEE-FLU derivatives (Figure 1), two hydroxyl groups are located, which were the main objects to the thermochemical theoretical investigations. The geometry optimization for these newly synthesized TFMPs and each of their chemical forms was performed twice: first, for the assumption that only the –OH(1) group undergoes a dissociation process, and second, for the assumption that only the –OH(2) group undergoes a dissociation process.

For the theoretical calculations, the AIMAll (Atoms in Molecules All) application (Toth) was used additionally. This application performs the calculations on the basis of the wave function, which was obtained using the GAUSSIAN98 calculation package and the CPCM/DFT/B3LYP/6-311++G (d,p) calculation method [8].

## 4. Conclusions

The DFT method was applied to the theoretical study of the newly synthesized TFMP derivatives with the two hydroxyl groups located in the side chain. The theoretical calculations showed that in the gaseous phase and aqueous medium, the hydroxyl group attached closer to the TFMP three-membered ring with the electron-withdrawing –CF_3_ group seems to be the chemically active place in the molecules of the studied compounds. From the thermochemical point of view, in the gaseous phase, the HHAT mechanism of releasing the hydrogen atom or a proton was preferred, while in the aqueous medium, the SET-PT or SPLET mechanism was preferred. Based on the values of the thermochemical parameters, the theoretical ability to scavenge free radicals by these new derivatives in the aqueous medium can be set in the following order: MAE-TPR > ABu-TPR > HEE-FLU. The QTAIM analysis of the new derivatives allowed identifying the intramolecular hydrogen bonds between the H-atom of the hydroxyl group and the N-atom of the side chain. Using the value of the potential energy density for the critical point of the bond path, the energy of these bonds was determined, which classified them as weak bonds.

Our results shed new light on the correlation of theoretical with experimental data for the studied compounds. Considering the theoretical calculations, the phenothiazines possess antioxidant properties. However, in the previous studies, we demonstrated the pro-oxidative properties of the same compounds in the in vitro system. We suppose that the observed pro-oxidative activity may be a consequence of the applied concentration of the compound, the incubation time, and the type of cell line used in the experiment. This hypothesis is in agreement with the literature data, which indicate both pro-and antioxidant properties of phenothiazines in the in vitro and in vivo experiments [37]. It is worth emphasizing that the metabolism of phenothiazines includes several oxidation processes which are catalyzed by peroxidases and lead to the formation of cation radicals. These cation radicals are supposed to be responsible for the pro-oxidant activity of phenothiazines. However, their stability may be limited by cellular events such as membrane association [38].

## Figures and Tables

**Table 1:** The values (kcal × mol^−1^) of the thermochemical parameters in the gaseous phase and in the aqueous medium for the hydroxyl groups located in the side chain of the investigated TFMP derivatives (the digit in parentheses identifies the given hydroxyl group in a molecule). The values (kcal × mol^−1^) of ∆BDE, ∆PDE, and ∆PA were calculated in the aqueous medium.

	FLU	APh-FLU	MAE-TPR	ABu-TPR	HEE-FLU
**Gaseous Phase**					
BDE (1)	99.1	–	92.1	98.5	98.0
BDE (2)	–	98.3	91.4	97.0	96.8
IP	155.6	156.5	161.5	160.6	158.2
PDE (1)	257.7	–	244.9	252.2	254.1
PDE (2)	–	251.6	244.2	250.7	252.9
PA (1)	369.6	–	340.9	356.5	360.0
PA (2)	–	348.7	341.5	349.6	355.8
ETE (1)	43.7	–	65.5	56.3	52.3
ETE (2)	–	59.4	64.2	61.6	55.3
**Aqueous Medium**					
BDE (1)	94.3	–	86.0	94.3	93.8
BDE (2)	–	92.8	84.6	90.6	93.1
IP	83.5	86.7	87.6	87.4	86.7
PDE (1)	53.3	–	38.7	47.1	47.4
PDE (2)	–	46.4	37.2	43.5	46.7
PA (1)	64.1	–	55.1	64.0	60.8
PA (2)	–	60.1	53.3	59.2	60.9
ETE (1)	72.6	–	71.2	70.5	73.3
ETE (2)	–	72.9	71.6	71.6	72.5
					
∆BDE	–	–	1.4	3.7	0.7
∆PDE	–	–	1.5	3.6	0.7
∆PA	–	–	1.8	4.8	0.1

**Table 2:** Calculated parameters (in atomic units) in gaseous and aqueous phases for the critical point of the bond path between hydrogen and oxygen atoms in the hydroxyl group: electron density ($\varrho_{BCP}$), Laplacian of electron density ($\nabla_{\varrho_{BCP}}^{2}$), potential energy density ($V_{BCP}$), kinetic electron density ($G_{BCP}$), total energy density ($H_{BCP}$).

Compound	Group	Bond Path between Atoms	$\varrho_{BCP}$	$\nabla_{\varrho_{BCP}}^{2}$	$V_{BCP}$	$G_{BCP}$	$H_{BCP}$
Gaseous Phase							
FLU	–OH(1)	O51–H52	0.36103	−2.47444	−0.76510	0.07325	−0.69185
APh-FLU	–OH(2)	O19–H34	0.35820	−2.46741	−0.76065	0.07190	−0.68875
MAE-TPR	–OH(1)	O26–H28	0.34402	−1.79570	−0.57436	0.06272	−0.51164
	–OH(2)	O27–H34	0.34110	−1.78820	−0.56957	0.06126	−0.50831
ABu-TPR	–OH(1)	O28–H44	0.36031	−2.46190	−0.76294	0.07373	−0.68921
	–OH(2)	O19–H37	0.36192	−2.48677	−0.76654	0.07242	−0.69412
HEE-FLU	–OH(1)	O34–H35	0.36657	−2.50894	−0.77544	0.07410	−0.70134
	–OH(2)	O25–H48	0.35682	−2.45795	−0.75789	0.07170	−0.68619
Aqueous Medium							
FLU	–OH(1)	O51–H52	0.35724	−2.45912	−0.75797	0.07160	−0.68637
APh-FLU	–OH(2)	O19–H34	0.35485	−2.45107	−0.75399	0.07061	−0.68339
MAE-TPR	–OH(1)	O26–H28	0.36003	−2.05718	−0.66478	0.07523	−0.58953
	–OH(2)	O27–H34	0.35527	−2.03688	−0.65751	0.07415	−0.58337
ABu-TPR	–OH(1)	O19–H37	0.36096	−2.48715	−0.76507	0.07164	−0.69343
	–OH(2)	O28–H44	0.35644	−2.45112	−0.75599	0.07161	−0.68439
HEE-FLU	–OH(1)	O34–H35	0.36475	−2.51694	−0.77333	0.07205	−0.70128
	–OH(2)	O25–H48	0.35250	−2.43066	−0.74919	0.07076	−0.67843

**Table 3:** Distance (*d*) between selected atoms in the gaseous phase and in the aqueous medium. Estimated value of H-bond energy (*E_HB_*). Calculated parameters (in atomic units) in gaseous and aqueous phases for the critical point of the bond path between the nitrogen atom of the side chain and the hydrogen atom of the given hydroxyl group: electron density ($\varrho_{BCP}$), Laplacian of electron density ($\nabla_{\varrho_{BCP}}^{2}$), potential energy density ($V_{BCP}$), kinetic electron density ($G_{BCP}$), total energy density ($H_{BCP}$).

Derivative	Atoms	*d* (Å)	*E_HB_* (kcal × mol^−1^)	Group	$\varrho_{BCP}$	$\nabla_{\varrho_{BCP}}^{2}$	$V_{BCP}$	$G_{BCP}$	$H_{BCP}$
Gaseous phase									
FLU	N38–H52	2.29	–	–	–	–	–	–	–
APh-FLU	N18–H34	2.22	−4.6	OH(2)	0.02037	0.07216	−0.01472	0.01638	0.00166
MAE-TPR	N22–H28	2.44	–	–	–	–	–	–	–
	N22–H34	2.32	–	–	–	–	–	–	–
ABu-TPR	N18–H44	2.24	–	–	–	–	–	–	–
	N14–H37	2.40	–	–	–	–	–	–	–
HEE-FLU	N28–H35	4.51	–	–	–	–	–	–	–
	N22–H48	2.18	−4.9	OH(2)	0.02164	0.07484	−0.01566	0.01718	0.00152
	O31–H35	2.45	–	–	–	–	–	–	–
Aqueous medium									
FLU	N38–H52	2.23	–	–	–	–	–	–	–
APh-FLU	N18–H34	2.17	−5.0	OH(2)	0.02211	0.07577	−0.01598	0.01746	0.00148
MAE-TPR	N22–H28	2.46	–	–	–	–	–	–	–
	N22–H34	2.25	–	–	–	–	–	–	–
ABu-TPR	N18–H44	2.19	−4.8	OH(1)	0.02138	0.07363	−0.01535	0.01688	0.00153
	N14–H37	2.39	–	–	–	–	–	–	–
HEE-FLU	N28–H35	4.54	–	–	–	–	–	–	–
	N22–H48	2.11	−5.7	OH(2)	0.02495	0.08212	−0.01834	0.01944	0.00110
	O31–H35	2.48	–	–	–	–	–	–	–

## Data Availability

Not available.
